# Modelling Systemic Iron Regulation during Dietary Iron Overload and Acute Inflammation: Role of Hepcidin-Independent Mechanisms

**DOI:** 10.1371/journal.pcbi.1005322

**Published:** 2017-01-09

**Authors:** Mihaela Enculescu, Christoph Metzendorf, Richard Sparla, Maximilian Hahnel, Johannes Bode, Martina U. Muckenthaler, Stefan Legewie

**Affiliations:** 1 Institute of Molecular Biology, Mainz, Germany; 2 Pediatric Oncology, Hematology & Immunology, University Hospital Heidelberg, Heidelberg, Germany; 3 Molecular Medicine Partnership Unit, Heidelberg University, Heidelberg, Germany; 4 Department of Gastroenterology, Hepatology and Infectious Diseases, Medical Faculty, University Hospital, Heinrich-Heine-University Düsseldorf, Düsseldorf, Germany; ETH Zurich, SWITZERLAND

## Abstract

Systemic iron levels must be maintained in physiological concentrations to prevent diseases associated with iron deficiency or iron overload. A key role in this process plays ferroportin, the only known mammalian transmembrane iron exporter, which releases iron from duodenal enterocytes, hepatocytes, or iron-recycling macrophages into the blood stream. Ferroportin expression is tightly controlled by transcriptional and post-transcriptional mechanisms in response to hypoxia, iron deficiency, heme iron and inflammatory cues by cell-autonomous and systemic mechanisms. At the systemic level, the iron-regulatory hormone hepcidin is released from the liver in response to these cues, binds to ferroportin and triggers its degradation. The relative importance of individual ferroportin control mechanisms and their interplay at the systemic level is incompletely understood. Here, we built a mathematical model of systemic iron regulation. It incorporates the dynamics of organ iron pools as well as regulation by the hepcidin/ferroportin system. We calibrated and validated the model with time-resolved measurements of iron responses in mice challenged with dietary iron overload and/or inflammation. The model demonstrates that inflammation mainly reduces the amount of iron in the blood stream by reducing intracellular ferroportin transcription, and not by hepcidin-dependent ferroportin protein destabilization. In contrast, ferroportin regulation by hepcidin is the predominant mechanism of iron homeostasis in response to changing iron diets for a big range of dietary iron contents. The model further reveals that additional homeostasis mechanisms must be taken into account at very high dietary iron levels, including the saturation of intestinal uptake of nutritional iron and the uptake of circulating, non-transferrin-bound iron, into liver. Taken together, our model quantitatively describes systemic iron metabolism and generated experimentally testable predictions for additional ferroportin-independent homeostasis mechanisms.

## Author Summary

The importance of iron in many physiological processes relies on its ability to participate in reduction-oxidation reactions. This property also leads to potential toxicity if concentrations of free iron are not properly managed by cells and tissues. Multicellular organisms therefore evolved intricate regulatory mechanisms to control systemic iron levels. A central regulatory mechanism is the binding of the hormone hepcidin to the iron exporter ferroportin, which controls the major fluxes of iron into blood plasma. Here, we present a mathematical model that is fitted and validated against experimental data to simulate the iron content in different organs following dietary changes and/or inflammatory states, or genetic perturbation of the hepcidin/ferroportin regulatory system. We find that hepcidin mediated ferroportin control is essential, but not sufficient to quantitatively explain several of our experimental findings. Thus, further regulatory mechanisms had to be included in the model to reproduce reduced serum iron levels in response to inflammation, the preferential accumulation of iron in the liver in the case of iron overload, or the maintenance of physiological serum iron concentrations if dietary iron levels are very high. We conclude that hepcidin-independent mechanisms play an important role in maintaining systemic iron homeostasis.

## Introduction

Iron is an essential element for the organism. It plays a critical role in oxygen transport, DNA synthesis, mitochondrial energy metabolism and as a cofactor of numerous enzymes [1, 2]. However, excess free iron catalyzes reactions that result in the formation of reactive oxygen species and oxidative stress. Hence, iron homeostasis must be maintained within a narrow range to provide sufficient iron for cellular function while preventing the generation of oxidative stress [3]. Systemic iron homeostasis is predominantly controlled by the interaction of the liver produced hormone hepcidin with its receptor, the iron transporter ferroportin (Fpn), resulting in the degradation of Fpn [4–7].

Fpn is the only known cellular iron exporter [8, 9]. It controls iron export from duodenal enterocytes that take up dietary iron, from iron-recycling macrophages, and from hepatocytes that store iron. Iron release from cells through Fpn requires the ferroxidases ceruloplasmin and/or hephaestin [10–12].

Hepcidin is produced in response to iron availability (via the BMP6/SMAD signaling pathway), erythropoetic demand (via erythroferrone), hypoxia and inflammatory mediators (via JAK/STAT signaling) [13–17]. Binding of hepcidin triggers Fpn internalization, ubiquitination and subsequent lysosomal degradation and thus causes iron retention in iron exporting cell types [18]. In addition, Fpn expression is regulated at the transcriptional level by hypoxia-inducible factor-2alpha (HIF2*α*) in response to hypoxia and iron deficiency [19, 20] as well as by BACH1 and Nrf2 in response to excess heme or oxidative stress [21]. At the translational level its expression is controlled by iron regulatory proteins (IRP), which bind to an iron responsive element (IRE) located in its 5’UTR [22, 23]. Furthermore, Fpn expression is controlled by miRNAs [24, 25].

Under physiological conditions serum iron is bound to the transport glycoprotein transferrin. Transferrin saturation is used as a measure for the serum iron level. The fact that hepcidin and Fpn expression are tightly regulated by numerous control mechanisms assures that physiological concentrations of transferrin-bound iron are maintained. Increased transferrin saturation increases hepcidin expression [26–28], leading to enhanced Fpn degradation and reduced duodenal iron absorption and iron mobilization from storage iron. In the case of hereditary hemochromatosis, where genetic perturbations cause hepcidin deficiency, chronic iron overload and organ damage will develop [29, 30]. On the other end of the spectrum, mutations in genes causing hepcidin overexpression will induce chronic iron deficiency and anemia [31].

Similarly, in an inflammatory setting, Fpn expression is decreased by hepcidin-dependent and hepcidin-independent mechanisms. While hepcidin expression is increased by inflammatory cytokines, such as IL6, causing Fpn degradation, Fpn transcription is additionally reduced [32–35]. Both control mechanisms reduce serum iron levels (hypoferremia), acting as an effective innate immunity mechanism that restricts access of microbes to iron [36]. However, if inflammation persists, the lack of iron results in a reduced iron supply for erythropoiesis, causing anemia of inflammation, which is frequently observed in hospitalized patients.

So far it is not clear how the different control mechanisms quantitatively impinge on Fpn to maintain cellular or systemic iron homeostasis. Studies in both, hepcidin knockout mice and mice with an engineered point mutation in Fpn that render it resistant to hepcidin binding (Fpn C326S mice) clearly demonstrate that LPS treatment results in a similar reduction of serum iron level compared to wild type mice [37, 38]. This indicates that hepcidin is dispensable for the generation of inflammation-induced hypoferremia under some conditions.

**Fig 1:**
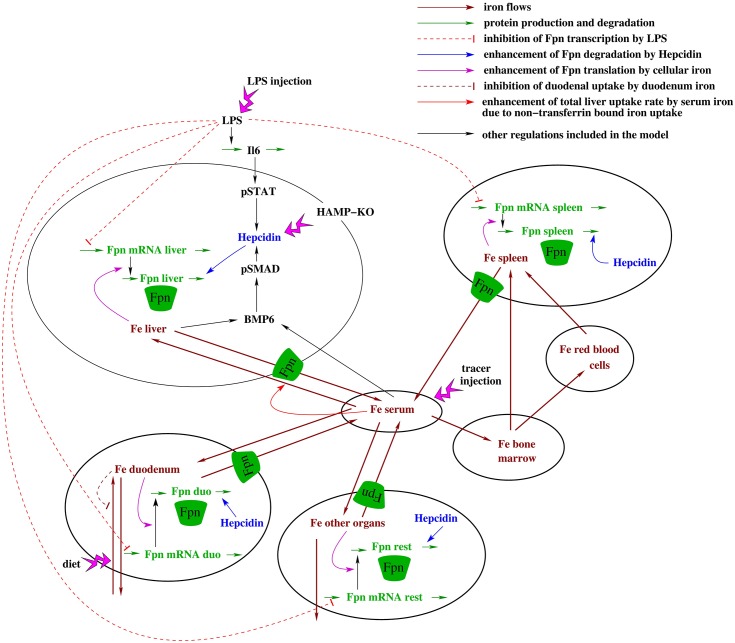
Scheme of the model including iron pools, iron fluxes and regulation of systemic iron levels by the hepcidin/ferroportin regulatory system. The iron content of seven compartments is described within the model. Besides serum, liver, spleen, bone marrow, red blood cells, and duodenum, the compartment ‘other organs’ accounts for the iron content of the remaining murine body. Iron absorption and loss in the duodenum and iron loss via skin and fur are considered. Iron export from the peripheral compartments into serum is regulated by ferroportin. The model accounts for inhibition of Fpn transcription by inflammation, regulation of Fpn translation by intracellular iron, as well as hepcidin-mediated post-translational destabilization of ferroportin protein. Transcriptional regulation of hepcidin by the BMP6/SMAD and IL6/STAT pathways are included in the model. Furthermore, the model considers regulation of BMP6 by liver and plasma iron.

**Fig 2:**
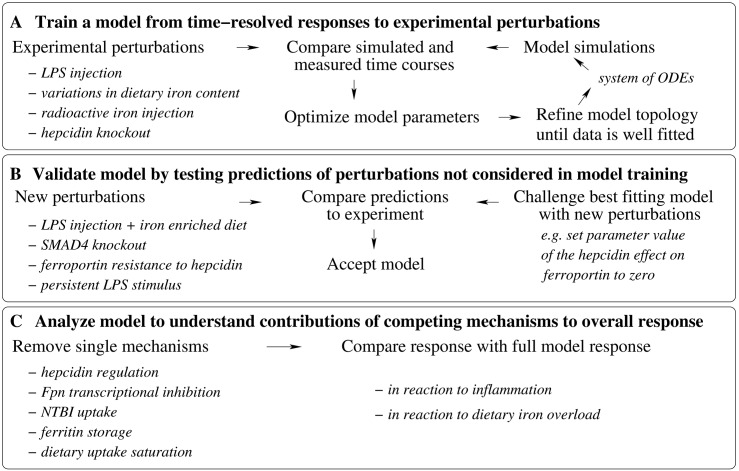
Summary of the different steps of the study. A-The mathematical model based on ordinary differential equations for the time dynamics of iron pools and regulating proteins was derived from the data. Starting from a core model topology, model parameters were found by fitting simulated time courses to experimental measurements of the systems response to different perturbations. The starting model topology was successively improved by including new regulatory mechanisms (e.g. regulation of iron flows by ferroportin, regulation of ferroportin by hepcidin) and the fitting cycle repeated until the model could fit well all of the data. B-The model prediction accuracy was tested for perturbations not used in training (new experiment in this study and previously published data not used in step A). The effect of the different perturbations was simulated by changing parameter values or initial conditions of the simulations. C-The model response to inflammation and dietary iron overload was analyzed to learn the quantitative contributions of the different mechanisms included to the overall response. Several mechanisms were removed one by one and the model simulations were compared to the results of the full model.

To dissect the quantitative contributions of hepcidin dependent post-translational and transcription-mediated Fpn control mechanisms under inflammatory conditions and to identify network components that contribute to the establishment of organ-specific iron pools, we generated a multi-scale model describing systemic iron homeostasis at the organ level. We extended our previously established model for hepcidin regulation via the BMP6/SMAD and the IL6/STAT3 signaling pathways [39] by now considering organ iron pools, organ-specific *Fpn mRNA* and protein synthesis/degradation rates and the impact of Fpn levels on the iron export from organs into blood (see Fig 1). The model was fit to own experimental data obtained in mice maintained on different iron diets or exposed to inflammatory stimuli (peritoneal LPS injection) as well as to previously published data by Lopes et al. [40], Daba et al. [41], Deschemin et al. [38] and Lesbordes et al. [42]. The model was validated by correctly predicting the responses of iron pools and iron-related proteins to a combined stimulus of dietary iron-loading and LPS treatment. Finally, we applied the model to assess the individual contributions of Fpn regulatory mechanisms to the response to dietary iron perturbations and inflammation and to analyze mechanisms for the establishment of organ specific iron-pools. Fig 2 gives an overview of the different steps of the study.

## Results

### Model derivation and implementation

Iron homeostasis in the body is maintained by intricate regulatory mechanisms of iron uptake, release and flux between compartments. Systems biological approaches are required to fully understand the dynamics of this complex network. So far mathematical models only described subsystems of iron metabolism, including liver iron metabolism [43, 44], intestinal iron absorption [45], iron release from macrophages [46] or storage of iron in ferritin [47]. To describe iron metabolism on a systemic level, Lopes et al. derived a whole-body model, which integrated iron fluxes between blood and different organs [40]. This model revealed how iron fluxes and distribution of iron pools between organs change upon alterations in dietary iron levels. However, the model by Lopes et al. lacked the underlying regulatory mechanisms responsible for maintenance of iron homeostasis and therefore it only described flux changes phenomenologically (by fitting each condition separately).

Motivated by this, we set out to develop a whole-body model of iron homeostasis, which explicitly describes intra- and extracellular regulatory loops that sense and modulate iron fluxes between different compartments. As a major means of regulation, we focused on the systemic regulation of iron metabolism by the hepcidin-Fpn regulatory axis (see Introduction). Our model allowed us to simultaneously fit time-resolved data for multiple experimental perturbations, and to dissect the impact of individual regulatory loops on systemic iron homeostasis.

The structure of our model is outlined in Fig 1. The concentration of iron in the non-cellular part of the blood stream (Fe serum) is the central hub. From the serum, iron can be imported into various organs. This raises the total intracellular iron pool in each organ (Fe liver, Fe spleen, Fe bone marrow, Fe red blood cells, Fe duodenum). Besides these major iron-containing organs, a substantial amount of iron can be found in the remaining body. We modeled this by including a lumped iron pool that sums up all remaining intracellular iron pools in the body (Fe other organs). In several reverse reactions, iron can also be exported from peripheral organs into the blood. Additionally, there is uptake of dietary iron in the duodenum and loss of iron from the duodenum and from the compartment ‘other organs’, which represent loss via the shedding of enterocytes and skin cells desquamation, respectively. Moreover, red blood cells receive iron from plasma through the bone marrow compartment and deliver iron into the spleen, e.g. into splenic macrophages that recycle iron from aging red blood cells. Additionally, iron uptake by spleen macrophages due to ineffective erythropoiesis is included [48].

The iron flux model initially described by Lopes et al. corresponds to the organs and iron fluxes (dark red arrows) depicted in Fig 1. In this study, we additionally considered that the export of iron from peripheral organs is controlled by the iron exporter Fpn which is predominantly located at the plasma membrane of three cell types: duodenal enterocytes, macrophages and hepatocytes. Fpn expression is described separately for each organ and is controlled by three regulatory mechanisms (see Introduction): (i) inflammatory cues (e.g. LPS) reduce the transcription of *Fpn mRNA*; (ii) intracellular iron levels enhance the translation of *Fpn mRNA* into protein; (iii) the turnover of Fpn protein is enhanced by the soluble polypeptide hepcidin.

Hepcidin expression is activated by the iron-sensing BMP6/SMAD pathway and by an inflammatory signaling cascade, which involves production of cytokines (primarily IL6 [49]) and the subsequent phosphorylation of STAT3 transcription factor in hepatocytes. Taken together, our model describes several auto-regulatory loops controlling iron homeostasis as well as multi-layered regulation by inflammatory signals, all of which converge on the modulation of Fpn expression levels.

To describe these phenomena, we derived a system of 20 coupled ordinary differential equations (see S1 Text). For example, the dynamics of the bone marrow iron pool derived from the model topology in Fig 1 is given by

$$\frac{d[Fe^{bm}]}{dt}=F_{ser\to bm}-F_{bm\to RBC}-F_{bm\to spl},$$

with *F*_*ser* → *bm*_, *F*_*bm* → *RBC*_ and *F*_*bm* → *spl*_ quantifying the inflow from serum into bone marrow and the outflows from the bone marrow into the RBCs and spleen compartments, respectively. The individual reaction rates were mainly described using mass action kinetics. For instance, it was assumed that most iron fluxes are proportional to the iron concentration in the originating compartment, e.g.

$$F_{ser\to bm}=v^{bm}\left[Fe^{ser}\right],\;F_{bm\to RBC}=v^{RBC}\left[Fe_{bm}\right]\;F_{bm\to spl}=v^{spl}\left[Fe_{bm}\right],$$ (1)

were the rate constants *v*^*bm*^, *v*^*RBC*^ and *v*^*spl*^ are model parameters (determined by fitting to the data, see next section). Iron export from peripheral organs into blood is additionally assumed to be proportional to the respective Fpn level, e.g.

$$F_{duo\to ser}=u^{duo}\left[Fe^{duo}\right]\left[Fpn^{duo}\right]$$

for the flow of iron from the duodenum into the serum. Thereby, we denote by *v*^*organ*^ the rate constants of iron import into the organs and by *u*^*organ*^ the rate constants of iron export into the serum. Fpn expression is described using a standard model of mRNA transcription and protein translation. Details about the Fpn regulation by LPS, iron and hepcidin can be found in S1 Text. Hepcidin induction by IL6 and BMP6 signaling pathways was described by a previously calibrated model [39], which considers signal integration on the hepcidin promoter using thermodynamic state ensemble approach (in the following referred to as ‘hepcidin promoter model’).

The computational model in SBML format is available in the Supplement (see S1 Model).

### Model simulation and calibration

The model contained many unknown parameters (e.g. rate constants of the iron flows between compartments *v*^*bm*^, *v*^*RBC*^, *v*^*spl*^ in Eq 1, see previous section), which had to be estimated by fitting the simulation results to experimental data. To reduce the complexity of this fitting problem, we assumed in some cases that homologous reactions in different compartments proceed with the same kinetic rate constants (see S1 Text). For instance, the degradation rate of *Fpn mRNA* and the Michaelis-Menten constant corresponding to *Fpn mRNA* inhibition by LPS is considered to be equal in all organs. Furthermore, the kinetic parameters of the hepcidin promoter model were fixed to the values that we had previously determined using systematic perturbations in the HuH7 cell culture system [39]. Nevertheless, 48 kinetic parameters remained to be estimated from the data. Additionally, 20 scaling parameters were fitted to match a model formulated in absolute concentration units to experimental data given in arbitrary units.

The model was calibrated based on time-resolved data from male C57BL/6-mice, which were either measured as a part of this study or taken from the literature (in total 344 data points). As summarized in Table 1 the calibration dataset comprised three experimental perturbations, which were either applied alone or in combination:

1. global alterations of body iron levels were introduced by changing the dietary iron content from age 4 to 9 weeks ([41] and this study);
2. the flux of iron through the compartments of the body was monitored by injecting small amounts of an iron tracer, and measuring its distribution in a time-resolved manner [40];
3. animals were challenged by an inflammatory stimulus (intra-peritoneal injection of LPS, [38] and this study).

**Table 1:** Experimental data used for model calibration. The table summarizes the experimental perturbations, time scales and measured quantities for the different datasets used.

mouse strain	experimental perturbation	Ref.	Fig.	output	time scale
C57BL/6	LPS injection	new data S1 Dataset	S1 Fig	serum iron, liver *hepcidin mRNA*, liver *BMP6 mRNA*, serum IL6, liver pSTAT, liver pSMAD, spleen Fpn mRNA, liver Fpn mRNA, liver Fpn protein	72 hours
C57BL/6	LPS injection	new data S2 Dataset	Fig 3	serum iron, liver iron, spleen iron, duodenum iron, RBC iron, liver *hepcidin mRNA*, liver *BMP6 mRNA*, liver Fpn mRNA, liver pSTAT, liver pSMAD, liver Fpn protein, spleen Fpn protein	48 hours
C57BL/6	high iron diet	new data S2 Dataset	Fig 6	serum iron, liver iron, spleen iron, duodenum iron, RBC iron, liver *hepcidin mRNA*, liver *BMP6 mRNA*, liver Fpn mRNA, liver pSTAT, liver pSMAD, liver Fpn protein, spleen Fpn protein	4 weeks
C57BL/6	reduced/increased dietary iron	[41] and S3 Dataset	S2 Fig	serum iron, liver iron, spleen iron, liver *hepcidin mRNA*, liver *BMP6 mRNA*	9 weeks
C57BL/6	radioactive iron injection	[40]	S3 Fig	tracer uptake in serum, liver, bones, duodenum, spleen, RBC, other organs	28 days
wt and Hep -/- C57BL/6	LPS injection/hepcidin knockout	[38]	S4 Fig	serum iron	6 hours
Hep -/- C57BL/6	hepcidin knockout	[42]	S4 Fig	liver iron	8 months

These experiments were mostly performed in wild type animals, but data from hepcidin knockout mice were also included. Model simulations were performed by analytically calculating a steady state before perturbing the system by a change in diet or in inflammatory status. The different experimental perturbations were mimicked in the model by changing parameter values or the initial conditions for the solving of the ordinary differential equations that describe the temporal dynamics of iron pools and regulatory proteins (see section ‘Model derivation and implementation’). For example, a change in dietary iron corresponds in the model to a change in the parameter value describing the influx on iron into the duodenum. Injection of LPS corresponds to a change in the initial condition for the LPS concentration to a nonzero value.

Iron pool sizes of compartments are given as *μg* per animal, with experimental data from individual mice scaled to a standard mouse weight of 25 *g* at 10 weeks of age. Details on the simulation of tracer iron distribution are given in S1 Text. Fitting of the simulated trajectories to the experimental measurements was done using a multi-start local optimizer and by minimizing the *χ*^2^ metric which is a weighed difference between model and data (S1 Text). The robustness of the model predictions was assessed by simulating them for multiple parameter combinations with a similar goodness-of-fit.

### The mathematical model of systemic iron homeostasis accurately fits perturbations in dietary iron levels and LPS-induced inflammation

We describe the dynamics of iron pools and regulatory proteins using a set of ordinary differential equations (see previous section and S1 Text). The kinetic parameters in this model were unknown and had to be estimated by fitting the model simulations to experimental data. To generate the data necessary for model calibration and for the testing of the fitted model, we performed time-resolved experimental measurements in mice. We subjected 9–10 weeks old male C57BL/6-mice to an intra-peritoneal LPS injection (1*μg*/*g* body weight) and followed the dynamics of iron-related parameters. At defined time points post injection (0.5/1/2/4/6/8/24/36/48/72 hours in a first experiment and 6/18/48 hours in a second experiment), we measured the levels of iron in serum, liver, spleen, duodenum and red blood cells. Complementary, we also fitted data of previous studies that analyzed temporal dynamics of whole-body iron metabolism in response to dietary iron content changes or upon injection of LPS or iron tracer [38, 40, 41].

**Fig 3:**
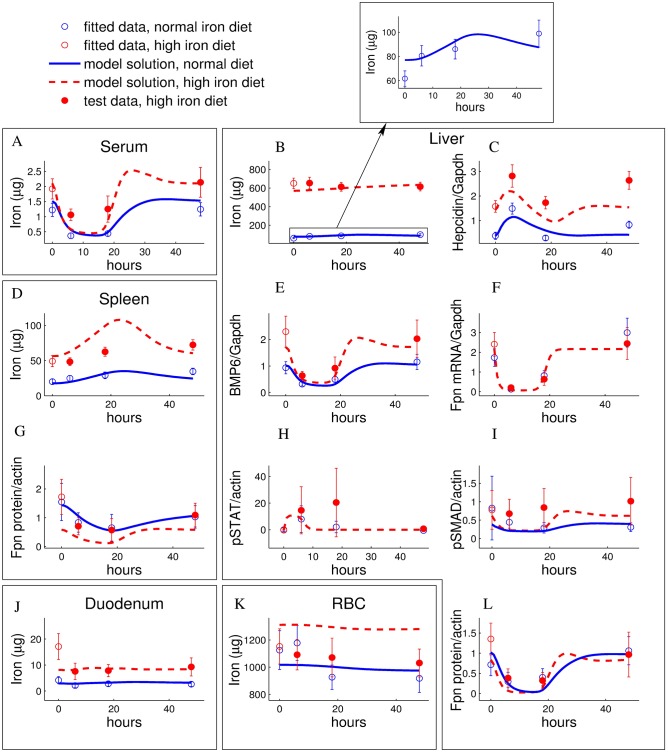
LPS-induced dynamics of iron-related parameters under normal/enriched iron diet is well reproduced/predicted by the model. A-Serum iron, B-Liver Iron, C-Liver hepcidin, D-Spleen iron, E-Liver BMP6, F-Liver Fpn mRNA, G-Spleen Fpn protein, H-Liver pSTAT, I-Liver pSMAD, J-Duodenum iron, K-Red blood cells iron, L-Liver Fpn protein. 4–6 weeks old male C57BL/6-mice were administrated a normal diet, containing 200 ppm iron (blue), or a high iron diet, supplemented by 2% carbonyl iron containing about 20000 ppm iron (red). After 4 weeks, mice were injected with 1 *μg* LPS/g body weight and sacrificed 6/18/48 hours after the injection. Experimental data are given as means with standard deviation of 4–6 replicates and the model simulation for the best fitting parameter set is represented by curves (solid lines: fitted time courses, dashed lines: predicted time courses). Data represented be empty circles were used in fitting as a part of the calibration data set (LPS response for normal diet and the iron parameters after 4 weeks of high iron diet before injection of LPS). The LPS response for high iron diet data (filled circles) was used to test the model predictions. See Materials and Methods for the description of the experiment.

LPS injection (1*μg*/*g* body weight) led to a transient drop in serum iron levels which was accompanied by iron accumulation in liver and spleen, but not in duodenum or red blood cells (see blue lines in Fig 3A, 3B, 3D, 3J and 3K and S1 Fig). In order to further characterize iron re-distribution, we quantified iron transport regulators in peripheral organs using qPCR and western blotting. The iron exporter Fpn was downregulated at the protein level in liver and spleen which likely explains iron accumulation in these compartments (blue lines in Fig 3G and 3L). Fpn downregulation is expected to be controlled by hepcidin, as we found inflammatory signaling pathways controlling hepcidin expression (serum IL6, liver pSTAT3) to be activated upon LPS injection (see Fig 3H and S1 Fig). Accordingly, we found liver *hepcidin mRNA* expression to be upregulated upon LPS injection (Fig 3C and S1 Fig). This most likely translates into increased levels of bioactive hepcidin in the circulation, as hepatic mRNA levels typically closely reflect the amount of released hepcidin peptides [50–52]. In addition, we also observed a strong downregulation of *Fpn mRNA* expression in liver and spleen (blue lines in Fig 3F and S1 Fig), as has previously been reported [33]. Hence, two independent pathways exist for Fpn regulation, a hepcidin-dependent post-transcriptional mechanism and a transcriptional mechanism that may be independent of hepcidin.

We fitted our mathematical model to the time-resolved measurements summarized in Table 1 using the procedure described in the Model section and S1 Text. The final model fits (Fig 3, blue lines and S1–S4 Figs) show a good quantitative match to the data: When averaged over all 344 data points (characterized by the means and standard deviation of 2–6 individual replicates), the best fits were about one standard deviation from the experimental data (*χ*^2^/*N* = 1.08). Furthermore, the model qualitatively reproduced the following key features of iron metabolism and accurately described their dynamics:

1. LPS injection induced iron accumulation in liver and spleen, but not in duodenum and red blood cells (Fig 3, blue lines).
2. Increased dietary iron content led to iron accumulation in liver and spleen, whereas the circulating serum iron levels increased only slightly and thus show homeostasis. A mild reduction in dietary iron affects none of these pools to a significant extent (S2 Fig).
3. The dynamics of a radioactive iron tracer applied to the mouse changed with the iron diet content [40]. The model simultaneously explained these data for three different dietary regimes (see S3 Fig).
4. Hepcidin knockout in mice led to increased serum iron levels and long-term liver iron accumulation (S4 Fig).

From this we concluded that our mechanistic model accurately describes iron homeostasis under various experimental conditions.

### Model correctly predicts iron homeostasis during LPS/dietary co-challenge, hepcidin resistance of ferroportin or loss of SMAD-signalling

We next challenged the model by testing its accuracy in predicting conditions not used for model calibration (data summarized in Table 2). First, we tested how our model would predict the LPS-response in iron-loaded animals, a condition where both STAT3 and SMAD1/5/8-signaling are altered. This was done by changing the value of the parameter that describes dietary iron uptake ([*Fe*_*food*_], see S1 Text) by the same fold-change as the experimental increase in dietary iron. Our model predicted that the LPS-induced dynamics of iron-related parameters for mice maintained on an iron-rich diet should be comparable to those maintained on a regular diet, albeit starting from a higher set point (red lines in Fig 3). Experimentally we maintained male C57BL/6-mice on an iron-rich diet (20000 ppm iron) containing 100 times more iron than the normal diet for 4 weeks, and subsequently injected a single dose of LPS (1*μg*/*g* body weight). The experimental data confirm the predictions of the model: the measured dynamics matched the model prediction for most variables (serum, liver and duodenum iron, liver hepcidin, *BMP6 mRNA*, liver pStat and pSmad, liver *Fpn mRNA* and protein). Deviations of the model fit from the experimental data were observed for diet-induced changes in the initial concentrations of spleen iron, RBC iron, spleen Fpn protein (Fig 3D, 3K and 3G). We concluded that our model reflects the main features of the response of iron metabolism towards LPS stimulations under iron overload conditions.

**Table 2:** Experimental data used for model validation. The table summarizes the experimental perturbations, time scales and measured quantities for the different datasets used.

mouse strain	experimental perturbation	Ref.	Fig.	output	time scale
C57BL/6	LPS injection after four weeks of high iron diet	new data	Fig 3	serum iron, liver iron, spleen iron, duodenum iron, RBC iron, liver *hepcidin mRNA*, liver *BMP6 mRNA*, liver Fpn mRNA, liver pSTAT, liver pSMAD, liver Fpn protein, spleen Fpn protein	48 hours
*Slc*40*a*1^*C*326*S*^	hepcidin-resistant Fpn knockin	[37]	Fig 4A	serum, liver, spleen iron	8 weeks
Smad4Co/Co;Alb-Cre	liver-specific SMAD4 knockout	[53]	Fig 4B	serum, liver, spleen iron and hepcidin	2 months

**Fig 4:**
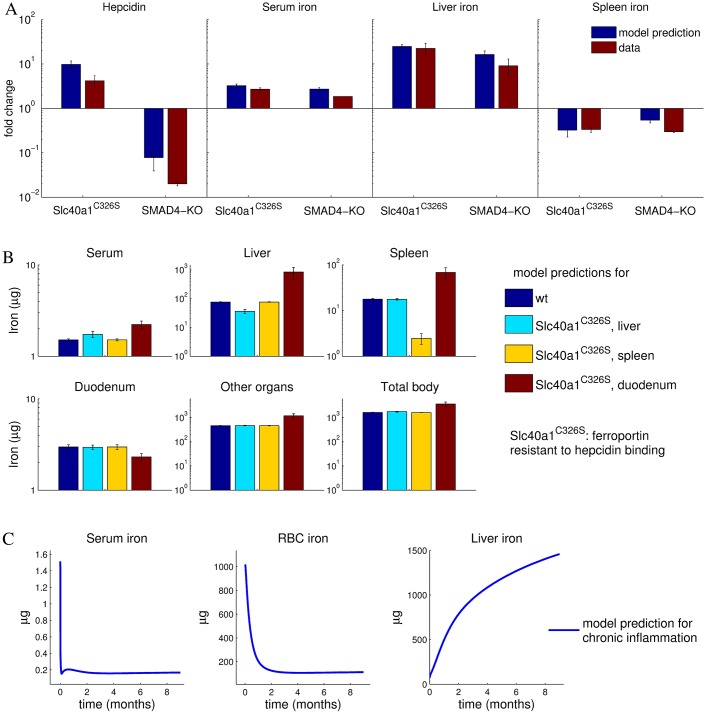
Model correctly predicts responses to perturbations in the SMAD4-hepcidin-pathway as well as development of anemia under chronic inflammation. A-Model quantitatively predicts experimentally measured responses for 2 months old C326S knock-in mice expressing a hepcidin-resistant FPN mutant or or SMAD4-knockout mice. The model simulations are shown as blue bars and the corresponding data from [37] and [53] as red bars, respectively. Fold changes are referred to the wildtype levels. The model error bars are calculated from the predictions of the 30 best fitting parameter sets (see S1 Text). B-Model prediction for body iron pools when ferroportin regulation by hepcidin is out of action in one of the indicated organs. Shown are model simulations whithout experimental validation. C-Model qualitatively reproduces the development of anemia of inflammation upon chronic elevation of body LPS. Simulation of plasma, RBC and liver iron evolution when the inflammatory Il6/STAT pathway is permanently activated by a persistent LPS stimulus (0.17 *μg*/g body weight). Shown are model simulations without a quantitative comparison to experiments.

To further validate the model, we challenged it with perturbations inside the regulatory network and compared the results to previously published data. Two experimental strategies have been performed to block the hepcidin negative feedback loop: First, disruption of Bmp6 signaling specifically in liver was achieved by using data derived from conditional SMAD4 knockout mice [53]. Second, a knockin mouse was established (*Slc*40*a*1^*C*326*S*^), which harbors a Fpn mutation that can no longer bind to hepcidin [37]. We reproduced these new conditions in the model by setting the SMAD expression level to zero (this has an influence on the hepcidin expression level calculated via the hepcidin promoter model, see Model section) or simultaneously setting the parameter values corresponding to the hepcidin effect on ferroportin degradation to zero ($k_{2}^{liver}$, $k_{2}^{duo}$, $k_{2}^{spleen}$, see Eq. 7 in S1 Text), respectively. In both cases, the model predictions, increased serum and liver iron pools and decreased spleen iron content (see Fig 4A), were confirmed by the experimental data [37, 53]. Moreover, as in the experimental data, Fpn resistance to hepcidin resulted in elevated hepcidin expression, whereas loss of SMAD-signaling resulted in a marked drop of hepcidin expression (Fig 4A).

We next explored the organ-specific role of Fpn regulation by hepcidin in more detail, by simulating the response of the system under the assumption that hepcidin-resistant Fpn is expressed in a tissue-specific manner. To this end, the parameter values corresponding to the hepcidin effect on ferroportin degradation ($k_{2}^{liver}$, $k_{2}^{duo}$, $k_{2}^{spleen}$, see Eq. 7 in S1 Text) were separately set to zero. Only the elimination of hepcidin-mediated Fpn regulation in the duodenum had a systemic effect on iron levels by increasing iron levels in serum, spleen, liver and the total body. By contrast, the simulation of hepcidin-resistant Fpn in liver or spleen resulted only in a local effect with a decreased iron pool in the respective organ and minimal changes in the other organs (Fig 4B). We conclude that hepcidin shows its strongest effect on steady state iron pools by regulating Fpn in the duodenum, where iron is taken up and lost. By contrast, Fpn level in liver and spleen predominantly affects the fraction of iron that circulates through these organs. Mouse models with tissue specific hepcidin-resistance have not been described so far. However, tissue-specific deletion of the FPN gene has been studied in *Fpn*^*flox*/*flox*^ mice carrying an intestine-restricted villin-Cre transgene that is inducible by tamoxifen [54]. Upon tamoxifen-induced expression, the Cre recombinase cuts out the intestinal FPN gene, and this leads to severe deficiency in the blood, liver and spleen [54]. This also indicates a systemic effect of duodenal Fpn levels on the body iron pools.

We further compared changes in the dynamics of iron-related parameters in response to chronic inflammation predicted by the model to qualitative knowledge from the literature. Chronic inflammation can cause anemia of inflammation, which is characterized by decreased serum iron and hemoglobin levels [55]. In this disease, inflammatory cytokines induce hepcidin expression, which results in reduced Fpn-mediated iron export, thus limiting iron availability in the blood stream for erythropoiesis in the bone marrow. If the inflammation persists anemia will develop as a consequence [56]. Chronic inflammation was mimicked in the model by assuming a constant source term in the equation that describes the time derivative of the LPS concentration (see Model section and Eq. 3 in S1 Text). Our model reflects the known iron-related alterations of chronic inflammation: during persistent inflammatory stimulation by LPS, serum iron levels decreased by about 85% within 2 days, while RBC iron decreased over a longer time scale until it finally stabilized at about 10% of the normal level after two months (Fig 4C). In agreement with data reported in [57], we find that the liver iron content increases in response to chronic inflammation (Fig 4C).

The above described model analyses, comprising both fitting and prediction, show that our mechanistic model is able to accurately describe a broad spectrum of perturbations on the quantitative level in many cases and on the qualitative level in most cases. Hence, our model allows us to mechanistically dissect systemic iron homeostasis and to evaluate to which extent hepcidin or Fpn contribute to the establishment of hypoferremia or maintenance of organ iron pools.

### Inflammation-induced hypoferremia is caused by a dynamic interplay of hepcidin-dependent and independent mechanisms regulating ferroportin expression

**Fig 5:**
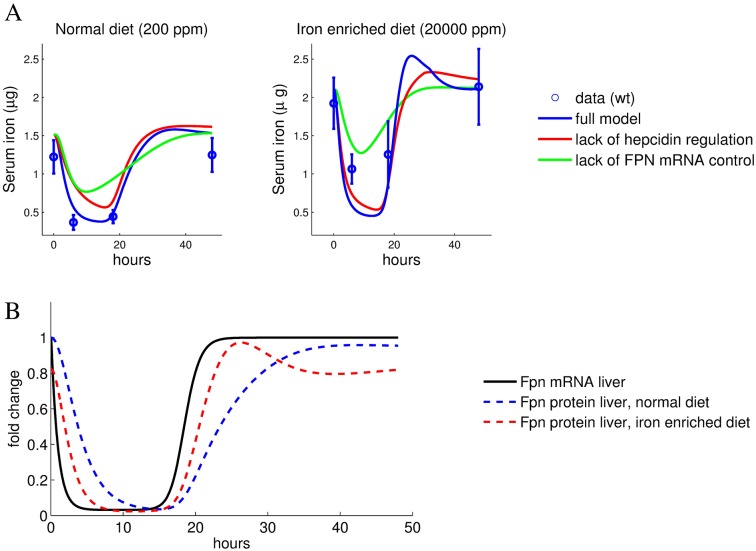
Hepcidin-mediated Fpn control and inhibition of Fpn transcription contribute to the acute inflammatory response. A-Experimental data (means with standard deviation) and simulated data (lines) for serum iron content following peritoneal injection of LPS for mice maintained on a standard or iron-enriched diet. Comparison of the full model with models where either hepcidin-mediated ferroportin degradation or inflammation-mediated ferroportin mRNA reduction are removed. The simulations correspond to the best fitting parameter set. B-LPS-induced changes of liver ferroportin mRNA and protein levels relative to the normal diet steady state.

Fpn is regulated on the transcript and protein level in response to inflammation [9], with hepcidin-dependent regulation considered to be the major contributor to hypoferremia. As our model encompasses both mechanisms of Fpn regulation (cell-autonomous transcriptional inhibition and hepcidin-mediated regulation), it allowed us to analyze the relative contribution of each mechanism separately by simulating the LPS response when either the transcriptional or the post-translational LPS effect on Fpn protein levels was eliminated. The initial condition of the simulations was the non-perturbed steady state of the system with both regulatory mechanisms included. Starting from this initial state, the reaction to LPS injection was simulated while keeping either hepcidin levels or *Fpn mRNA* levels constant (the time derivatives of these model variables were set to zero, see Eqs. 1 and 6 in S1 Text). Interestingly, the lack of the LPS mediated hepcidin induction showed an almost normal drop in serum iron levels (75% of the complete model, see Fig 5A, red line). By contrast, the removal of the Fpn transcriptional control in response to LPS had a stronger effect and alleviated hypoferremia to 50% of the control (see Fig 5A, green line). The strong effect of inflammation-mediated transcriptional regulation of Fpn became even more evident in animals with dietary iron overload. Removal of hepcidin regulation in this case led to a near to normal level of inflammation-induced hypoferremia, while the serum iron drop induced by hepcidin regulation alone was reduced (Fig 5A). This shows that hypoferremia arises by a combination of hepcidin-dependent and independent mechanisms with the total effect on the serum iron level being less than additive.

Even though the relative contribution of transcriptional control of Fpn expression seems dominant over the hepcidin mediated control of Fpn in the high-iron setting, hepcidin does play a critical role. The increased basal level of hepcidin in iron loaded animals reduces the Fpn protein half-life (via the terms *k*_2_[*hep*], see Eq. 7 in S1 Text and S6 Fig). This in turn couples Fpn protein levels more tightly to *Fpn mRNA* levels (compare red and blue lines in Fig 5B). As a result, induction of hypoferremia by transcriptional inhibition of Fpn and the recovery of serum iron levels are faster in iron loading conditions, with and without inflammatory control of hepcidin (Fig 5A).

From this, we conclude that the transcriptional control of Fpn expression is the major determinant of the degree of hypoferremia. It amplifies the effect of hepcidin-mediated protein degradation in an acute inflammatory setting.

### The ferroportin/hepcidin regulatory axis fails to explain iron accumulation in the liver

**Fig 6:**
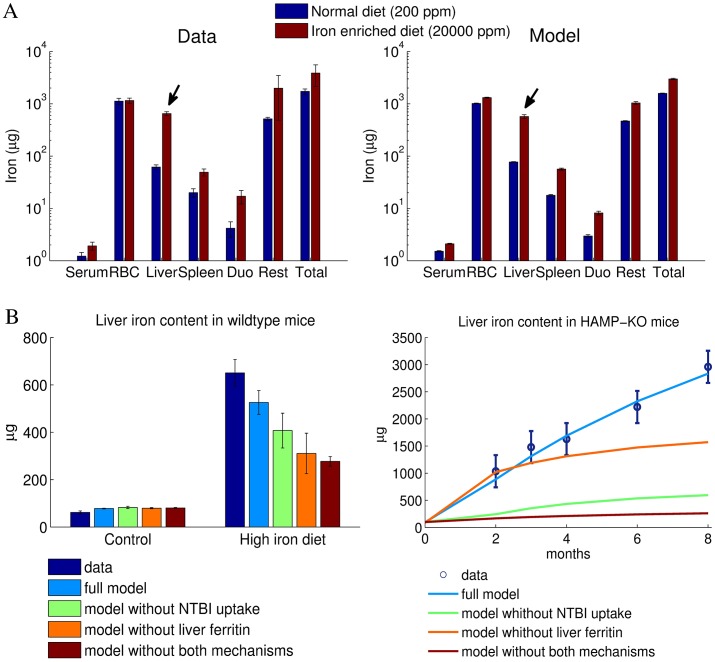
Iron overload as a consequence of an iron enriched diet leads to the preferential iron accumulation in the liver, which is quantitatively reproduced by the model considering NTBI uptake and liver ferritin storage. A-The measured distribution of iron between the organs is reproduced by the model fits for both normal and an iron enriched diet. The iron content of all compartments increases in mice maintained for 4 weeks on an iron rich diet, with most iron accumulating in the liver (arrows). B-Measured liver iron content under conditions of dietary iron overload and in HAMP-KO mice as well as best fit for the full model and models lacking NTBI uptake or liver ferritin storage, or both.

We next focused our analysis on iron distribution among body compartments under conditions when mice were maintained either on an iron-enriched diet or on a regular diet. Our data reflect the role of the liver as the main iron storage organ of the body, which is well described in the literature [58, 59]. Fig 6A shows the measured and fitted distributions of iron between the different pools in mice fed a normal or iron-enriched diet, respectively (same experiment/data as in Fig 3). The iron content in all compartments except for the red blood cells increased after 4 weeks of dietary iron overload. The liver shows both the highest relative and absolute change of iron content: a 10-fold increase in liver iron levels corresponding to an additional ∼600*μg* iron per mouse (arrows in Fig 6A). The model fits these data quantitatively.

We experimentally checked whether liver iron accumulation correlates with a low expression of the iron exporter Fpn. Unexpectedly, hepatic Fpn protein levels were increased in mice fed with an iron-enriched diet, even though the BMP6/Hepcidin pathway was activated as expected (S5A Fig). Increased Fpn protein levels can be explained only partially by transcriptional regulation, as we observed only a modest increase in *FPN mRNA* levels (S5A Fig). While the macrophage marker F4/80 was increased in livers of LPS treated animals, this was not observed for iron-loaded animals compared to animals on a normal diet (S5B Fig), suggesting that increased hepatic Fpn protein levels in iron-loaded animals are not explained by the infiltration of macrophages in the liver that express higher levels of Fpn compared to hepatocytes. Thus, our data may suggest that the IRE/IRP-mediated translational regulation of Fpn expression is more pronounced than hepcidin-mediated Fpn regulation in the case of sustained dietary iron overload.

These experimental findings indicate that hepatic iron accumulation cannot be explained by hepcidin controlled Fpn degradation. As a matter of fact, the increase in Fpn levels should counteract iron accumulation. Hence, liver specific iron uptake must be assumed to be considerably higher to make up for the increased iron export. To investigate the cause for the increase in liver iron levels, we tested additional Fpn-independent regulatory mechanisms for the iron exchange between liver and serum and performed model selection based on their fitting performance. Two mechanisms have been included in the final model variant (these were also present in all simulations shown above).

1. Since the liver is able to store iron in a Fpn inaccessible form (e.g. ferritin, see [60, 61]), which cannot be directly exported back into the blood, we have restricted the pool of iron that can be exported from liver into the blood for high liver iron levels. This was implemented in the model by assuming that iron export from the liver to the serum is proportional to the liver iron content only up to a maximum value (see Model section and Eq. 10 in S1 Text). Liver iron exceeding this maximal value is assumed to be in a ferritin-bound form and thus inaccessible for transfer to the plasma (in the following referred to as ‘ferritin storage mechanism’).
2. While serum iron is bound to transferrin under physiological conditions (see Introduction), high iron diet may lead to accumulation of non-transferrin bound iron (NTBI) in the serum. Cells of the bone marrow mainly import transferrin bound iron via transferrin receptor 1 (TfR1), but the liver is able to absorb also non-transferrin bound iron (NTBI) through the zinc transporter ZIP14 [62]. We found that TfR1 expression was decreased in livers of iron-fed mice (see S5A Fig). Thus, transferrin bound iron uptake by the liver cannot explain liver iron accumulation. In agreement with previous studies of mice with genetically caused iron overload [63–65], we therefore assumed that the binding capacity of transferrin is saturated in iron loaded animals and NTBI is found in the serum. Since the absorption of NTBI in the liver occurs at a much higher rate than the transferrin mediated import [66], the final model variant considered that liver iron uptake becomes more pronounced when serum iron levels are elevated, as preferentially NTBI is taken up (see Eq. 9 in S1 Text, in the following referred to as ‘NTBI uptake mechanism’).

Fig 6B shows for the full model and models lacking one or both of these mechanisms the fitting performance of liver iron accumulation upon dietary iron overload and in HAMP-KO mice. We find that the full model is the only one that fits all liver iron data. We conclude that the hepcidin-Fpn axis alone is not sufficient to explain liver iron accumulation during dietary iron overload.

### Saturation of duodenal iron uptake is important for systemic iron homeostasis

Chronic iron overload causes irreversible organ damage as exemplified by disease conditions such as hereditary hemochromatosis [67]. Our own data and published data [14, 41] show that even pronounced increases in dietary iron levels translate into a comparably small accumulation of iron in organs (iron homeostasis). Specifically, we observed that a 100-fold increase in dietary iron content results in a less than two-fold increase in most body iron pools. The most dramatic change with a ∼10-fold increase occurs in the liver (see arrows in Fig 6A). Thus, all pools respond to dietary changes in a (much) less than proportional manner, both in vivo and in the model (see Fig 6A).

To better understand the mechanisms that maintain iron homeostasis, we systematically perturbed each parameter of the model (e.g. protein synthesis/degradation rates, iron import/export rates) and analyzed which one shows the most pronounced impact on serum iron levels and induces the measured response of body iron pools to changes in the diet (see S7 Fig). This analysis reveals that limited iron uptake in the duodenum (see below) is the most critical mechanism to buffer all body iron pools against increases in dietary iron.

**Fig 7:**
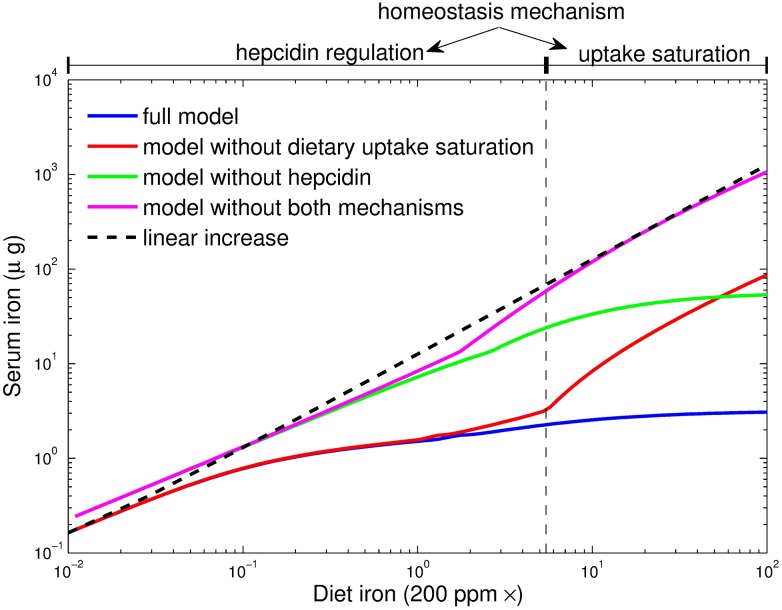
Hepcidin-mediated control and dietary uptake saturation are critical parameters for serum iron homeostasis under dietary iron overload. Simulation of steady state serum iron levels as a function of the dietary iron content for different model variants. For comparison, a linear increase of serum iron levels with increased dietary iron content is depicted.

Apical uptake of iron from the lumen into duodenal enterocytes occurs via the divalent metal iron transporter 1 (DMT-1). This process is regulated locally by cellular iron levels and hypoxia [68]. Under iron rich conditions, iron absorption into enterocytes decreases due to downregulation of iron transporters by the IRE/IRP system and hypoxia-inducible factor 2 [59, 69–71]. Based on this, we assumed in the model that iron uptake from the diet into duodenal enterocytes shows saturation (this mechanism was also present in all simulations shown above). This was implemented by using a Michaelis-Menten type equation to describe the duodenal iron uptake rate as a function of the dietary iron content (see Eq. 14 in S1 Text). The Michaelis-Menten constant of this equation (parameter *K*_*duo*_, see S1 Text) was fitted to approximately five times the normal iron diet content which results in strong saturation for the 100-times iron enriched diet. Eliminating this saturation by assuming a linear dependence of the absorption rate on the dietary iron content led to a loss of homeostasis for increasing dietary iron content (compare blue and red lines in Fig 7).

The model predicted that a 100-fold increase in dietary iron should result in a 2.5-fold increase in dietary iron uptake (40 and 100 *μg* iron per day in mice under normal and iron overload conditions, respectively). We have indirectly tested this prediction by determining the iron contents in individual organs and the remaining carcass and summing up the individual pools for mice on normal and high iron diets (Fig 6A). In line with the hypothesis of iron-uptake saturation, we found that raising the iron diet content by 100-fold caused an only 3-fold increase in total body iron levels.

Our modeling analysis showed that the saturated uptake mechanism does not explain homeostasis upon a reduction of dietary iron content (red and blue lines in Fig 7). We therefore analyzed the impact of other mechanisms and found that hepcidin was required for homeostasis at low and normal dietary iron. Elimination of hepcidin from the model was implemented by setting the hepcidin effect on ferroportin protein degradation to zero (parameters $k_{2}^{liver}$, $k_{2}^{duo}$, $k_{2}^{spleen}$ and $k_{2}^{rest}$ in Eq. 7 of S1 Text). Homeostasis in the range of low and normal dietary iron content is lost upon elimination of hepcidin from the model (green line in Fig 7). Furthermore, upon combined elimination of both mechanisms (uptake saturation and hepcidin regulation) homeostasis is lost over the full range of iron concentrations.

Thus, we conclude that both, regulated iron uptake by mechanisms operational in the duodenal enterocyte, and hepcidin-controlled responses that integrate information about systemic iron availability, are critical for stabilizing whole-body iron levels over a broad range of dietary iron concentrations.

## Discussion

### Model limitations

Even though the experiments used for model training comprise measurements perturbations in several parts of the assumed regulatory network, uncertainties exist in the parameter estimation and thus in the predictive power of the model. The model parameters could be better constrained by directly determining additional parameters or by increasing the sampling rate of the time course measurements, thereby reducing the degrees of freedom during the fitting process. However, direct measurements of iron transport rates or synthesis/degradation rates of regulatory proteins (e.g. FPN, DMT1 or ZIP14) or IRE/IRP binding activities are complex and would have gone beyond the purpose of this study.

For ethical reasons, measurements were restricted to a minimum number of time points that allow an evaluation of the time dynamics, since additional sampling would have required the sacrifice of more animals (e.g. 12 more animals for each time point for the diet+LPS experiment).

Due to these limitations, not all model parameters could by determined with the same precision (see S1 Text for the uncertainty interval of each parameter value). For example, the available data allow a good estimation of the hepcidin degradation rate, with values between 0.067 and 0.07 *h*^−1^ within the 30 best fitting parameter sets obtained using a local multi-start optimization strategy (see Calibration in S1 Text). In contrast, only the magnitude order can be estimated for the degradation rate of *BMP6 mRNA*, since its value varies between 1 and 9.5 *h*^−1^ within the same 30 best fitting parameter sets. To avoid uncertainty, we have ensured the robustness of the model predictions by assessing the simulation results for these 30 parameter values combinations that fit best the data, and did not restrict ourselves to the best fitting parameter set.

The analysis of the quantitative impact of different mechanisms in the model simulations showed that some regulations (e.g., hepcidin effect on ferroportin) are crucial for the systems response in certain conditions, but play only a supporting role for other experimental conditions (see previous sections). However, all variables and regulatory interactions included in the model were selected because they significantly improved the overall fitting of the calibration data set. Nevertheless, the model topology was chosen as simple as possible in view of the studied experimental conditions, since our aim was not to build a complete, but rather a minimal model that is able to describe these conditions. Thus, several regulatory mechanisms of iron metabolism described in the literature have been omitted (e.g. regulation of hepcidin during inflammation via activin B [33, 72]; ceruloplasmin/hephaestin for iron export by ferroportin [11, 12]; negative regulation of hepcidin via erythroferrone [73]). Even though they were apparently not critical for the reproduction of the experimental measurements, some of these mechanisms could also have been active in the experimental perturbations included in the study. Not including them in the model may explain some of the quantitative differences still present between model fits/predictions and measurements. The same holds for the explicit modeling of sub-organ compartments (such as Kupffer cells, hepatocytes, endothelial cells and stellate cells in liver), that have been omitted to keep the model as simple as possible and because their experimental acquisition was not feasible.

### Future studies

Our model predicted that hypoferremia during acute inflammation requires transcriptional downregulation of ferroportin mRNA, whereas post-transcriptional regulation via hepcidin plays a lesser role. This finding could be directly validated experimentally by ablating the inflammatory signaling branch controlling hepcidin expression. For instance, LPS injection could be performed in hepatocyte-specific STAT3 knockout mice [74] or in a knock-in mouse model in which hepcidin is expressed under the control of a heterologous promoter [75]. Additionally, transcriptional regulation of ferroportin could be studied in more detail using bioinformatics analyses of promoter sequence motifs and reporter gene assays in cell culture. Several transcription factors have been implicated in transcriptional regulation of ferroportin, including hypoxia inducible factor 2 (HIF2*α*), nuclear factor erythroid (Nrf2), metal-regulatory transcription factor 1 (MTF-1) and estrogen receptor [76]. Identification of the regulatory mechanisms that predominate in vivo would allow interfering with transcriptional regulation of ferroportin during inflammation and hence lead to a better understanding of its importance in inflammation, host-pathogen interactions and cancer.

According to our model, changes in dietary iron are mainly compensated by the BMP/SMAD/hepcidin negative feedback loop. This mechanism of iron homeostasis is well-known and the model predictions could be validated quantitatively by testing how changing dietary iron affect circulating iron levels in transgenic mice which express hepcidin under control of a heterologous promoter [75]. Taken together, our model represents a tool that may help to guide the design and interpretation of future experiments.

### Conclusions

We succeeded in establishing the first mechanistic model that explains the distribution of iron between pools, predicts accurately the responses of iron-loaded mice to stimulation by LPS, and evaluates quantitatively the roles of Fpn and hepcidin during the establishment of hypoferremia. The model supports the important role of hepcidin for the inflammatory response but shows that transcriptional regulation of Fpn after an inflammatory insult is required additionally to efficiently establish hypoferremia.

We also provided insights into the mechanisms that maintain iron homeostasis in response to alterations in the dietary iron content. Our results suggest that non-transferrin bound iron uptake by the liver (presumably through ZIP14) and hepatic iron storage in ferritin are the critical determinants for liver-specific iron accumulation during iron loading conditions. Finally, the model shows that regulation of duodenal iron uptake in an enterocyte intrinsic manner is as important as hepcidin-mediated regulation for the establishment of serum iron homeostasis under dietary iron overload conditions.

## Materials and Methods

### Ethics statement

All mouse breeding and animal experiments were approved by and conducted in observation of the guidelines of the EMBL Institutional Animal Care and Use Committee.

### Mouse experiments

#### Time and LPS dependent response of iron parameters

Male C57BL6/J mice were delivered by Janvier Labs, Saint-Berthevin Cedex, France. They were housed at the laboratory animal resource facility of the university of Düsseldorf under a constant 12h light-dark cycle and had ad-libitum access to food and water. At an age of 8 to 12 weeks mice were treated with LPS as described earlier in this article. After the indicated points in time mice were anesthesized and blood was drawn from the vena cava. Blood clotted at room temperature for 30 min before it was centrifuged at 10000 g for 10 min. Supernatant was collected and centrifuged again. Serum was collected as supernatant of the second centrifugation step and stored at -20°C. Measurement of serum interleukin 6 levels were determined using the multiplex technology xMAP (Luminex corp.) using the MILLIPLEX MAP Mouse Cytokine/chemokine Magnetic Bead Panel (Millipore) as indicated in the manufacturers manual.

#### Time, LPS and food-iron dependent response of iron parameters

For the LPS plus iron-diet experiment male C57BL6/J mice were used. Animals were housed at the laboratory animal resource facility at the EMBL under a constant light-dark cycle and had ad-libitum access to food (Teklad 2018S, Harlan, 200 ppm iron) and water. At 5 weeks of age 36 mice were put on control diet (SNIFF, EF E15510-24 +200 mg/kg iron) and 36 mice were put on a high-iron diet (SNIFF, EF E15510-24 + 2% carbonyl iron), which differed only in the iron content from the control diet. After 4 weeks on these diets, animals were split into LPS-treatment groups and control groups. LPS-treatment groups were treated with 1 ug LPS per g body weight by peritoneal injection with 100 ug LPS (Lipopolysaccharides purified by gel-filtration chromatography from Escherichia coli 0111:B4, product L3012, Sigma-Aldrich) per 1 mL 0.9% NaCl (pyrogen free NaCl injection solution, prod 2350756, BRAUN). Control animals were injected with the respective volume of NaCl only. After the indicated times animals were anesthetized and killed by terminal bleeding. Whole blood was collected in lithium heparin vials (prod. 41.1503.005, SARSTEDT) and used to acquire hematological parameters before generating plasma samples that were stored at -80°C until analysis. All organs (order of collection: liver, spleen, kidney, heart, lungs and duodenum) were removed and immediately snap-frozen in liquid nitrogen and then stored at -80°C until further analysis. The carcasses consisting of skin, fur, muscle, bones and the head, excluding the digestive tract to avoid contamination with food-iron, were stored at -20°C.

### Experimental procedures for data of published articles used in this model

The following data from male mice of the “food iron treatment” group (high, normal and low iron) from [41] were used: organ iron (liver & spleen), serum iron, transferrin saturation, gene expression in liver (Hamp1, Bmp6, Smad7, liver Id1). The data from this article was supplemented with western blot analysis (liver: beta-Actin, pSMAD1/5/8, pSTAT3; spleen: beta-Actin, Fpn1, Ferritin L) and qPCR (spleen: Ireg1/Fpn1, Tfr1) carried out in our lab.

Tracer iron levels in C57BL6 wild-type mice maintained on an iron-deficient, iron-adequate, or iron-loaded diet were taken from [40] (Organ Fe59 contents scaled to the whole mouse body). The data from all tissues not explicitly considered here (intestine, muscle, heart, fur, lungs, testis, kidneys, fat, stomach and brain) was summed up and used to fit the compartment ‘other organs’ in our model. Data corresponding to one time point *t* was rescaled with the same factor for all compartments, such that the overall tracer sum is equal to 100exp(−*rt*). In [40], a fixed iron lost rate *r* of 0.5% per day was used as derived from literature. Here, the iron lost rate *r* was assumed to be a model parameter, whose value has been determined by fitting the data.

### Haematology and tissue and plasma iron measurements

Hematological parameters were determined from blood collected in heparin vials (Sarstedt) using the ABC ScilVet analyzer (ABX Diagnostics). Tissue non-heme iron was measured in dried tissue samples using acid extraction and bathophenantroline (Sigma) as chromogen as described by [77]. Iron content of carcasses was determined in the same way with the following modifications: carcasses were ground in a mortar after drying, acid extraction was performed with the complete material from each carcass and iron measurement was performed on duplicate subsamples of the cleared extract. Plasma/serum iron was measured using the bathophenantroline-based method of the SFBC kit 80008 (BIOLABO, France) adapted to the 96-well format by scaling to 40 *μL* serum/plasma sample volumes [78]. Unsaturated iron binding capacity (UIBC) in plasma/serum was determined using the UIBC kit 97408 (BIOLABO, France) adapted to 96-well format by scaling to 20 *μL* serum/plasma sample volumes [37].

### qPCR measurements

RNA extraction cDNA synthesis and qPCR RNA was purified from tissue samples using Trizol reagent (life technologies) according to the manufacturers protocols with one additional ethanol-wash of RNA prepared from liver to reduce high A230nm. 2 ug total RNA were reverse transcribed using random hexamers and RevertAid (Fermentas). qPCR was performed on a StepOne thermocycler (Applied Biosystems) using the SYBR-green Master Mix (Applied Biosystems) and primers listed in S1 Text. Relative mRNA expression was calculated by the delta Ct method and normalized to the reference genes *Gapdh* or *beta-Actin* as indicated.

### Western blot

Snap-frozen tissues were homogenized in RIPA buffer supplemented with protease inhibitors (Roche) and protein concentrations were determined using the DC protein assay (BioRad). Total protein (60 ug) was subjected to western blot analysis with the antibodies listed in S1 Text. Western blots were imaged and analyzed using the Fusion-FX system (Vilber Lourmat). Protein levels were normalized by the level of beta-Actin.

## Supporting Information

S1 Text Supplementary text. Description of the mathematical model and fitting strategy, list of parameter values, list of primers used for qPCR, and list of antibodies used for western blot.
(PDF)

S1 Fig LPS injection leads to a serum iron drop as a result of hepcidin-dependent Fpn regulation and transcriptional inhibition of Fpn. 9–10 weeks old male C57BL/6-mice were injected intraperitoneally with 1 *μg* LPS/g body weight and sacrificed at 0.5/1/2/4/6/8/24/36/48/72 hours after the injection. Experimental data are displayed as means with standard deviation and the model solution for the parameter set that fits best all data from the calibration set is represented by lines.
(EPS)

S2 Fig Time-dependent responses in mice to reduced or enriched dietary iron content. The iron content of the food of 4-week old male C57BL/6-mice was reduced from 225 mg/kg to 100 mg/kg (red) or increased (blue) with 2% carbonyl iron corresponding to 19600 mg/kg for the indicated times after which mice were sacrificed [41]. Experimental data from [41] are displayed as means with standard deviation and the model solution for the best fitting parameter set is represented by curves.
(EPS)

S3 Fig Time-resolved radioactive iron uptake into body compartments for standard nutrition (blue), iron-reduced (green) and iron-enriched diet (red). Adult male C57BL6 mice on a diet containing 180 mg iron/kg, 6 mg iron/kg or 25 g iron/kg were injected with ^59^Fe and sacrificed at different time points within 4 weeks after the injection [40]. The ^59^Fe content of different organs was measured and the percent of the initial dose of radioactive tracer found in the body compartments included in the model was calculated. Experimental data from [40] are displayed as means with standard deviation and the model solution for the best fitting parameter set is represented by curves. Major quantitative differences between the three dietary conditions are found in liver (increased relative uptake with iron content) and red blood cells compartment (decreased relative uptake with iron content).
(EPS)

S4 Fig Serum and liver iron concentration in hepcidin knockout mice. A-Serum iron concentration and the response to LPS (2 mg/kg) 6h after injection in normal and hepcidin knockout mice. Experimental data from [38] and solution of best fitting model. B-Age dependent liver iron accumulation in hepcidin knockout mice between 2 and 8 month of age. Experimental data from [42] is shown as means with standard deviation and solution of the best fitting model is displayed as a curve.
(EPS)

S5 Fig Hepatic protein expression in response to normal and iron enriched diet. A-Despite elevated *BMP6* and *hepcidin mRNA* expression in the iron loaded mice, the ferroportin level is increased. TfR1 expression is decreased in mice maintained on an iron-enriched diet. B-Relative *F4/80 mRNA* levels in livers of mice on either diet and after peritoneal LPS-treatment or a combination of both as indicated in the figure legend.
(EPS)

S6 Fig Degradation rate of liver ferroportin protein after injection of LPS for normal and iron enriched diet. Model simulations corresponding to the time courses in Fig 3 show that iron overload increases the degradation rate of ferroportin protein in the liver. This corresponds to a reduction of ferroportin protein half-life upon iron overload.
(EPS)

S7 Fig Sensitivity of the different iron levels and of their changes following dietary iron overload to the different parameters included in the model. The map represents the quantities *m*_*ij*_ = *d* log *x*_*j*_/*d* log *p*_*i*_, where *x*_*j*_ are iron pools (left map) or dietary induced fold changes (right map), and *p*_*i*_ are the model parameters labeled on the y-axis.
(EPS)

S1 Model Computational model in SBML format. The file contains the model species, reactions between species including kinetic laws, best fitting parameter set, as well as initial conditions for each species corresponding to the steady state of the system for the given parameter values.
(XML)

S1 Dataset LPS injection dataset. The file contains measured values for several iron parameters in the first 72 hours after LPS injection in C57BL/6-mice. These data is part of the calibration data set and is shown in S1 Fig.
(XLSX)

S2 Dataset LPS injection + iron diet data set. The file contains measured values for several iron parameters in the first 48 hours after LPS injection in C57BL/6-mice. Results for both mice on a normal diet and on an iron enriched diet (4 weeks before LPS injection) are included. Data for the normal iron diet as well as the state after 4 weeks of iron enriched diet but before LPS injection were used for calibration. Remaining data (LPS response for iron enriched diet) were used for validation. All data are shown in Fig 3.
(XLSX)

S3 Dataset Iron diet data set. The file contains the part of the calibration data set shown in S2 Fig. These data were provided by the authors of Daba et al. 2012 [15].
(XLSX)
