# Supplementary material for: Modelling Systemic Iron Regulation during Dietary Iron Overload and Acute Inflammation: Role of Hepcidin-Independent Mechanisms
Source: PLoS Comput Biol. 2017 Jan 9;13(1):e1005322. doi: 10.1371/journal.pcbi.1005322 (PMC5261815; doi:10.1371/journal.pcbi.1005322)
Supplement: S1 Text — Description of the mathematical model and fitting strategy, list of parameter values, list of primers used for qPCR, and list of antibodies used for western blot. (PDF) [file pcbi.1005322.s001.pdf]

Supplementary information for "Systemic Iron Regulation during Dietary Iron Overload and Acute Inflammation"

Mathematical model

In the following, we describe the mathematical equations used to quantify hepcidin expression, ferroportin regulation, as well as the dynamics of the different iron pools included in the model (see Fig. 1 in the main text).

Hepcidin expression

The model is based on a previously published model for the hepcidin expression upon BMP6 and IL6 regulation [2], that gives us the concentration of hepcidin mRNA in the liver:

$$\frac{d[hep]}{dt} = r^{hep}_{synthesis} - r^{hep}_{degradation} = f([Bmp6],[Il6]) - k^{hep}_{deg}[hep], \tag{1}$$

where the function f is identical to the model in [2]. The concentration of Bmp6 entering Eq. 1 is assumed to be proportional to BMP6 mRNA, whose concentration is described by:

$$\begin{aligned}\frac{d[Bmp6]}{dt} &= r^{Bmp6}_{synthesis} - r^{Bmp6}_{degradation} \\ &= v^{Bmp6}_{max}\frac{[Fe^{liv}]}{K^{Bmp6}+[Fe^{liv}]}\min([Fe^{ser}],[Tf]) - k^{Bmp6}_{deg}[Bmp6].\end{aligned} \tag{2}$$

Thereby, we assume that liver iron content as well as transferrin-bound iron increase expression of Bmp6. $[Tf]$ is a model parameter that determines the maximal amount of iron that can be bound by transferrin.

The concentrations of LPS, Il6 mRNA and serum Il6 are described by:

$$\frac{d[LPS]}{dt} = -k^{LPS}_{deg}[LPS], \tag{3}$$

$$\frac{d[Il6_{mRNA}]}{dt} = \frac{[LPS]}{[LPS]+K^{Il6_{mRNA}}} - k^{Il6_{mRNA}}_{deg}[Il6_{mRNA}], \tag{4}$$

and

$$\frac{d[Il6]}{dt} = k^{Il6}_{synth}[Il6_{mRNA}]^4 - k^{Il6}_{deg}[Il6], \tag{5}$$

where the final $[Il6]$ gives the concentration entering the hepcidin promoter module in Eq. 1.

Ferroportin regulation

Ferroportin transcription in liver, spleen, duodenum and rest body is modeled by:

$$\begin{aligned}
\frac{d[Fpn^{liv}_{mRNA}]}{dt} &= \frac{1}{1+\frac{K_1^{liv}[Il6]}{K_2+[Il6]}} - k_{deg}^{Fpn_{mRNA}}[Fpn^{liv}_{mRNA}],\\
\frac{d[Fpn^{spl}_{mRNA}]}{dt} &= \frac{1}{1+\frac{K_1^{spl}[Il6]}{K_2+[Il6]}} - k_{deg}^{Fpn_{mRNA}}[Fpn^{spl}_{mRNA}],\\
\frac{d[Fpn^{duo}_{mRNA}]}{dt} &= \frac{1}{1+\frac{K_1^{duo}[Il6]}{K_2+[Il6]}} - k_{deg}^{Fpn_{mRNA}}[Fpn^{duo}_{mRNA}],\\
\frac{d[Fpn^{rest}_{mRNA}]}{dt} &= \frac{1}{1+\frac{K_1^{rest}[Il6]}{K_2+[Il6]}} - k_{deg}^{Fpn_{mRNA}}[Fpn^{rest}_{mRNA}],
\end{aligned} \tag{6}$$

with the inclusion of LPS-mediated inhibition in the synthesis of ferroportin mRNA. Thereby, K_2 is the Michaelis-Menten constant for the effect of $[IL6]$ on the Fpn_{mRNA} level, and K_1 is the maximal strength of the inhibition, assumed to be organ-specific. Ferroportin translation is then modeled by

$$\begin{aligned}
\frac{d[Fpn^{liv}]}{dt} &= k_{synth}^{Fpn^{liv}}(1+k_1^{liv}[Fe^{liv}])[Fpn^{liv}_{mRNA}] - k_{deg}^{Fpn^{liv}}(1+k_2^{liv}[hep])[Fpn^{liv}],\\
\frac{d[Fpn^{spl}]}{dt} &= k_{synth}^{Fpn^{spl}}(1+k_1^{spl}[Fe^{spl}])[Fpn^{spl}_{mRNA}] - k_{deg}^{Fpn^{spl}}(1+k_2^{spl}[hep])[Fpn^{spl}],\\
\frac{d[Fpn^{duo}]}{dt} &= k_{synth}^{Fpn^{duo}}(1+k_1^{duo}[Fe^{duo}])[Fpn^{duo}_{mRNA}] - k_{deg}^{Fpn^{duo}}(1+k_2^{duo}[hep])[Fpn^{duo}],\\
\frac{d[Fpn^{rest}]}{dt} &= k_{synth}^{Fpn^{rest}}(1+k_1^{rest}[Fe^{rest}])[Fpn^{rest}_{mRNA}] - k_{deg}^{Fpn^{rest}}(1+k_2^{rest}[hep])[Fpn^{rest}],
\end{aligned} \tag{7}$$

by considering the positive regulation of the translation rate by the respective organ iron content and enhancement of the degradation rate by hepcidin.

Dynamics of the iron pools

Finally, the seven iron pools are modeled by taking into account the different flows between the compartments and the ferroportin effect on the flows into the serum. In the following, rate constants for the ferroportin independent flows are denoted by v while u is used for the proportionality constant in ferroportin-dependent flows.
The dynamics of liver iron are given by

$$\frac{d[Fe^{liv}]}{dt} = F_{ser\to liv} - F_{liv\to ser} \tag{8}$$

with $F_{ser\to liv}$ and $F_{liv\to ser}$ quantifying the flows from serum into the liver and the backflow into the serum, respectively. Since the proportion of non-transferrin bound iron (NTBI) increases with increasing serum iron concentration $[Fe^{ser}]$, and because NTBI has a higher uptake rate than transferrin-bound iron [1], the flow of iron from the serum into the liver is a pice-wise linear function of the serum iron concentration. Specifically, we assume for the flow from serum into the liver

$$F_{ser\to liv} = v_1^{liv}\min([Fe^{ser}], th) + v_2^{liv}\max([Fe^{ser}] - th, 0) \tag{9}$$

with the lower rate constant v_1^{liv} for $[Fe^{ser}] < th$ (transferrin-bound iron) and the upper rate constant $v_2^{liv} > v_1^{liv}$ for $[Fe^{ser}] > th$ (NTBI).

The flow from liver into the serum is given by

$$F_{liv \rightarrow ser} = u^{liv} \min([Fe^{liv}], [Fe^{liv}_{max}])[Fpn^{liv}], \tag{10}$$

beeing therefore proportional to the ferroportin level in the liver and up to a maximal value $[Fe^{liv}_{max}]$ proportional to the liver iron content. This function describes the fact that the liver iron content that exceeds the maximal value $[Fe^{liv}_{max}]$ is found in ferritin form, and thus non-available to be transferred to the plasma any more.
A similar assumption is made also for the spleen, leading to the following dynamic equation:

$$\frac{d[Fe^{spl}]}{dt} = v_1^{spl}[Fe^{RBC}] + v_2^{spl}[Fe^{bm}] - u^{spl} \min([Fe^{spl}], [Fe^{spl}_{max}])[Fpn^{spl}], \tag{11}$$

where v_1^{spl} and v_2^{spl} are the rate constants of the flows from RBCs and bone marrow into the spleen, and $u^{spl}[Fpn^{spl}]$ is the rate constant of the flow from the spleen into the serum.
The dynamics of the iron content in the bone marrow $[Fe^{bm}]$ are further given by:

$$\frac{d[Fe^{bm}]}{dt} = v^{bm}[Fe^{ser}] - (v^{RBC} + v_2^{spl})[Fe^{bm}], \tag{12}$$

with v^{bm}, v^{RBC} and v_2^{spl} beeing the rate constants of the flows from serum into bone marrow, from bone marrow into RBCs and from bone marrow into the spleen, respectively.
The temporal evolution of the red blood cell compartment is then given by

$$\frac{d[Fe^{RBC}]}{dt} = v^{RBC}[Fe^{bm}] - v_1^{spl}[Fe^{RBC}]. \tag{13}$$

Similar equations describe the evolution of the duodenum iron content and the iron content of the rest body, whith input and output flows in the duodenum and an output from the rest body, simulating iron uptake from food and iron lost via duodenum, skin and fur, respectively:

$$\frac{d[Fe^{duo}]}{dt} = v^{duo}_{max} \frac{[Fe_{food}]}{[Fe_{food}] + K_{duo}} \frac{1}{[Fe^{duo}]} + v^{duo}[Fe^{ser}] - u^{duo}[Fe^{duo}][Fpn^{duo}] - v^{duo}_{lost}[Fe^{duo}], \tag{14}$$

$$\frac{d[Fe^{rest}]}{dt} = v^{rest}[Fe^{ser}] - u^{rest}[Fe^{rest}][Fpn^{rest}] - u^{rest}_{lost} \min([Fe^{rest}], [Fe^{rest}_{max}]). \tag{15}$$

Thereby, we consider a Michaelis-Menten term for the uptake of iron from food and further assume that this uptake decreases with increasing iron content in the duodenum. For the iron lost via skin and fur, a limiting maximal value $u^{rest}_{lost}[Fe^{rest}_{max}]$ is presumed.
The evolution of the serum iron concentration is finally given by

$$\frac{d[Fe^{ser}]}{dt} = F_{input} - F_{output}, \tag{16}$$

with the input from organs given by

$$\begin{aligned} F_{input} &= u^{liv} \min([Fe^{liv}], [Fe^{liv}_{max}])[Fpn^{liv}] + u^{spl} \min([Fe^{spl}], [Fe^{spl}_{max}])[Fpn^{spl}] \\ &\quad + u^{duo}[Fe^{duo}][Fpn^{duo}] + u^{rest}[Fe^{rest}][Fpn^{rest}] \end{aligned}$$

and the output into organs given by

$$F_{output} = v_1^{liv} \min([Fe^{ser}], th) + v_2^{liv} \max([Fe^{ser}] - th, 0) + (v^{bm} + v^{duo} + v^{rest})[Fe^{ser}].$$

Model simulation and fitting

Simulation of experimental perturbations

For a given set of model parameters, a steady state could be calculated analitically for all variables of the model by setting their time derivatives to zero. Experimental perturbations were simulated by correspondingly perturbing this steady state.
LPS-injection was mimicked by setting the value of the LPS concentration from 0 (steady state) to 1. A change in the iron diet was performed by adapting the parameter $[Fe_{food}]$ according to the dietary iron content used in a particular experiment. Hepcidin knockout was simulated by setting the hepcidin concentration $[hep]$ to zero. Ferroportin resistance to hepcidin binding was mimicked by setting the hepcidin-dependent degradation rate k_2 to zero in one organ (organ specific resistance), or all organs (general resistance), respectively. For the SMAD4-knockout, the BMP6 concentration (downstream of SMAD4) entering the function $f([Il6], [BMP6])$ in Eq. 1 was set to zero. A chronic inflammation was simulated by introducing a constant production term in Eq. 3.
For the simulation of the tracer experiment, a reduced model containing only the iron pools was considered. For each dietary condition, the flow rates between the iron compartments were first calculated using the full model and assumed to hold proportionately also for the tracer experiment, since the tracer amount injected was very small compared to the total body iron (0.01%). For the nonlinear fluxes (flows from serum into liver, liver to serum and spleen to serum), the flow of the full model was linearized and the linear rate was used when simulating the tracer iron distribution with the reduced model.
Finally, for the analysis of the relative contribution of hepcidin regulation and ferroportin inhibition in LPS-mediated hypoferremia, the dynamics of the model was simulated by holding either the hepcidin level or the Fpn mRNA levels constant after LPS injection, at the values corresponding to the unperturbed steady state.

Calibration strategies

Based on the ODE system, the time courses of the state variables after each experimental perturbation were calculated and compared to the experimental data. The SUNDIALS CVode package and ODEMEX (a CVode wrapper for Matlab) were used for solving the initial value problems. Parameter fitting was based on minimizing the χ^2 metric, given by $\chi^2 = (M_i - D_i)^2/\sigma_i$, where M_i, D_i and σ_i are the simulated value, the measured value and the experimental error, respectively. Parameter optimization was done using the trust-region-reflective algorithm in Matlab (lsqnonlin). In order to circumvent local minima, the model was repeatedly fitted to the data starting from 10000 quasi randomly distributed parameter sets. The model parameters and their values corresponding to the best fit are summarized in Tables 1,2. The best fit yielded a value of $\chi^2 = 372$ for the $N = 344$ data points. The standard deviation for the model predictions in Figs. 3, 5 and S4 Fig was calculated by simulating the best 30 models with a χ^2-value between 372 and 400.

Parameter values

As stated in the main manuscript and shown in Fig. 2 and S1 Fig-S4 Fig, the time courses of the six experiments considered could be fitted simultaneously, so that the model mimics all data using one unique parameter set. In particular, the same flow rates of iron were used for the steady-state transport of normal iron between compartments and the distribution of radioactive iron. One exception had to be made: the flow rate of iron from RBC into spleen, for which separate parameter values were fitted to the tracer experiment and all other data sets. The reason for this is that elimination of RBCs has two components: age-dependent elimination and random,

age-independent elimination [3]. In the short time period of the tracer experiment only the random-elimination is captured, because older RBCs do not contain radioactive iron. By constrast, both components are active in all other experiments. Thus, our combined datasets and model allow the prediction of the magnitude of random elimination. Based on the optimal fits, the random elimination of RBC accounts for approximately 30% of the total elimination rate of RBCs, since the fitted value for the spleen iron uptake from RBC (v_1^{spl}) was about 3 times lower for the tracer experiment, compared to all other data sets (see Table 2).

Supplementary tables

Table 1. Model parameters

Parameter	Description	Units	Best fit val.	Min./max. val.
k_{deg}^{hep}	Hepcidin degradation rate	h^{-1}	0.07	0.067/0.07
v_{max}^{Bmp6}	Bmp6 maximal synthesis rate	h^{-1}	31.5	14.2/126.5
K^{Bmp6}	Michaelis-Menten constant Bmp6 synthesis	μg	19.6	16.5/55.7
k_{deg}^{Bmp6}	Bmp6 degradation rate	h^{-1}	2.4	1.0/9.5
k_{deg}^{LPS}	LPS degradation rate	h^{-1}	5.9	5.9/5.9
$K^{Il6_{mRNA}}$	Michaelis-Menten constant $Il6_{mRNA}$ synthesis	μg	2.6×10^{-6}	$2.6\times10^{-6}/2.6\times10^{-6}$
$k_{deg}^{Il6_{mRNA}}$	$Il6_{mRNA}$ degradation rate	h^{-1}	0.28	0.28/0.28
k_{synth}^{Il6}	Il6 synthesis rate	h^{-1}	646	136/872
k_{deg}^{Il6}	Il6 degradation rate	h^{-1}	4.45	4.45/4.45
K_2	constant Fpn_{mRNA} production	a.u.	0.0013	0.0003/0.002
$k_{deg}^{Fpn_{mRNA}}$	Fpn_{mRNA} degradation rate	h^{-1}	1.08	1.02/1.2
K_1^{liv}	constant Fpn_{mRNA}^{liv} production	a.u.	30.7	28/32.5
K_1^{spl}	constant Fpn_{mRNA}^{spl} production	a.u.	33.3	33.1/34.5
K_1^{duo}	constant Fpn_{mRNA}^{duo} production	a.u.	0.61	0.56/0.79
K_1^{rest}	constant Fpn_{mRNA}^{rest} production	a.u.	11.2	7.8/43.7
$k_{synth}^{Fpn^{liv}}$	Fpn^{liv} synthesis rate	h^{-1}	0.13	0.07/0.14
k_1^{liv}	constant Fpn^{liv} production	μg^{-1}	0.003	0.002/0.006
$k_{deg}^{Fpn^{liv}}$	Fpn^{liv} degradation rate	h^{-1}	0.055	0.01/0.06
k_2^{liv}	constant Fpn^{liv} degradation	a.u.	2.57	2.11/12.93
$k_{synth}^{Fpn^{spl}}$	Fpn^{spl} synthesis rate	h^{-1}	0.023	0.015/0.027
k_1^{spl}	constant Fpn^{spl} production	μg^{-1}	0.014	0.005/0.028
$k_{deg}^{Fpn^{spl}}$	Fpn^{spl} degradation rate	h^{-1}	0.003	0.0007/0.0038
k_2^{spl}	constant Fpn^{spl} degradation	a.u.	11.5	9.2/73.7
$k_{synth}^{Fpn^{duo}}$	Fpn^{duo} synthesis rate	h^{-1}	0.03	0.01/0.25
k_1^{duo}	constant Fpn^{duo} production	μg^{-1}	0.16	0.06/0.49
$k_{deg}^{Fpn^{deg}}$	Fpn^{duo} degradation rate	h^{-1}	0.021	0.0056/0.147
k_2^{duo}	constant Fpn^{duo} degradation	a.u.	1.43	0.78/4.16

Table 2. Model parameters (continued)

Parameter	Description	Units	Best fit val.	Min./max. val.
$k_{synth}^{Fpn^{rest}}$	Fpn^{rest} synthesis rate	h^{-1}	0.005	0.004/0.108
k_1^{rest}	constant Fpn^{rest} production	μg^{-1}	0.11	0.004/0.152
$k_{deg}^{Fpn^{rest}}$	Fpn^{rest} degradation rate	h^{-1}	0.029	0.025/0.129
k_2^{rest}	constant Fpn^{rest} degradation	a.u.	11.6	3.7/38.1
v_1^{liv}	low liver iron uptake	μg/h	3.96	2.78/9.81
v_2^{liv}	high liver iron uptake	μg/h	56.9	44.9/377.5
th	threshold serum iron value	μg	2.68	2.08/3.
u^{liv}	liver iron export rate	h^{-1}	0.08	0.05/0.19
$[Fe_{max}^{liv}]$	threshold value liver iron export	μg	119	100/159
v_1^{spl}	spleen iron uptake rate from RBC	h^{-1}	0.004	0.004/0.005
v_1^{spl}	spleen iron uptake rate from RBC, tracer experiment	h^{-1}	0.002	0.001/0.002
v_2^{spl}	spleen iron uptake rate from bones	h^{-1}	0.01	0.008/0.019
u^{spl}	spleen export rate	h^{-1}	0.24	0.21/0.36
$[Fe_{max}^{spl}]$	threshold value spleen iron export	μg	88	57/95
v^{bm}	bone marrow uptake rate	h^{-1}	2.83	2.66/4.16
v^{RBC}	RBC uptake rate	h^{-1}	0.058	0.055/0.075
v_{max}^{duo}	maximal duodenal uptake from food	μg/h	9.86	6.77/11.98
K_{duo}	saturation parameter duodenal uptake	μg/h	177	120/261
v^{duo}	duodenal uptake rate from blood	h^{-1}	0.7	0.6/1.3
u^{duo}	duodenal export rate	h^{-1}	0.88	0.66/1.38
v^{rest}	other organs uptake rate	h^{-1}	6.32	5.4/10.
u^{rest}	other organs export rate	h^{-1}	0.017	0.014/0.03
v_{lost}^{duo}	iron lost rate duodenum	h^{-1}	0.092	0.001/0.32
u_{lost}^{rest}	iron lost rate, rest	h^{-1}	0.003	0.002/0.004
$[Fe_{max}^{rest}]$	limit value, iron lost rest	μg	511	510/947

Table 3. List of primers used for qPCR.

gene name		5´ sequence 3´
Gapdh	F	TGTCCGTCGTGGATCTGAC
	R	CCTGCTTCACCACCTTCTTG
36b4	F	GCGACCTGGAAGTCCAACTA
	R	ATCTGCTGCATCTGCTTGG
beta-Actin	F	GCTTCTTTGCAGCTCCTTCGT
	R	ACCAGCGCAGCGATATCG
Tfr1	F	CCCATGACGTTGAATTGAACCT
	R	GTAGTCTCCACGAGCGGAATA
Smad6	F	GTTGCAACCCCTACCACTTC
	R	GGAGGAGACAGCCGAGAATA
Smad7	F	GCAGGCTGTCCAGATGCTGT
	R	GATCCCCAGGCTCCAGAAGA
BMP6	F	ATGGCAGGACTGGATCATTGC
	R	CCATCACAGTAGTTGGCAGCG
Hamp1	F	ATACCAATGCAGAAGAGAAGG
	R	AACAGATACCACACTGGGAA
Zip14	F	TGGAACCCTCTACTCCAACG
	R	CTGAGGGTTGAAGCCAAAAG
F4/80	F	GGAGGACTTCTCCAAGCCTATT
	R	AGGCCTCTCAGACTTCTGCTT

Table 4. Antibodies used for western blot.

protein	antibody	company	dilution	blocking/1st antibody
beta-Actin	mouse monoclonal	Sigma	1:20 000	5% milk
Ferroportin(non-boild SDS-sample)	rabbit affinity purified, MTP11-A Lot 548586A 10-L	Alpha Diagnostic int. (Biotrend)	1:500	5% milk
phospho-STAT3 (Tyr705)	mouse monoclonal; 9138 Lot 5	Cell Signaling	1:500	5% milk
phospho-SMAD1/5 (Ser463/465) SMAD8 (Ser426/428)	rabbit affinity purified; 9511 Lot 14	Cell Signaling	1:500	5% BSA
Ferritin L	goat affinity purified; sc-14420	Santa Cruz	1:200	5% milk
	α goat HRP		1:5 000	
	α mouse HRP		1:10 000	
	α rabbit HRP		1:5 000	

TBS 0.1 % Tween was used troughout. In the case of Ferritin, 0.01% SDS was added in addition. Blocking was done for 1h at room temperature, 1st antibodies were incubated at 4 °C overnight, except for the Actin-antibody (20 min room temperature).

References

1. Anderson GJ, Frazer, DM. Hepatic iron metabolism. Seminars in Liver Disease 2005; 25: 420-432.

2. Casanovas G, Banerji A, d'Alessio F, Muckenthaler MU, Legewie S. A Multi-Scale Model of Hepcidin Promoter Regulation Reveals Factors Controlling Systemic Iron Homeostasis. PLOS Computational Biology 2014;10: e1003421.

3. Lopes TJS, Luganskaja T, Spasić MV, Hentze MW, Muckenthaler MU, Schümann K, Reich JG. Systems analysis of iron metabolism: the network of iron pools and fluxes. BMC Systems Biology 2010;4: 112.
